# Statin in the treatment of patients with myocardial infarction: A meta-analysis

**DOI:** 10.1097/MD.0000000000010167

**Published:** 2018-03-23

**Authors:** Xue Han, Yi Zhang, Lin Yin, Lin Zhang, Yue Wang, Hongshan Zhang, Bo Li

**Affiliations:** Department of Cardiac Function, Second Affiliated Hospital of Kunming Medical University, Kunming, Yunnan, China.

**Keywords:** meta-analysis, myocardial infarction, statin

## Abstract

The purpose of this meta-analysis is to investigate whether statin is a key therapy for myocardial infarction (MI) by comparing all randomized controlled trials that appraised the effects of statin on risk of MI.

Pubmed, Embase, and Medline databases (up to December 2016) were used to search all related articles. Using the data from 18 available publications, we examined the efficacy in treating or reducing the risk of MI by using random-effects models of odds ratio (OR) comparing the highest with the lowest category.

Statins have demonstrated efficacy in treating or reducing the risk of MI (OR = 0.73, 95% confidence interval = 0.58–0.93, *P* = .010).

This meta-analysis suggests that statin have light efficacy in treating or reducing the risk of MI patients.

## 1 Introduction

The Global Status Report states that cardiovascular disease (CAD) has caused more and more deaths.^[1]^ Myocardial infarction (MI) is the most serious and fatal result of CAD. The main nosogenesis is the extensive necrosis of cardiomyocytes caused by prolonged ischemia.^[2]^ The development of CAD is a long-time process that suffered from erosion of endothelium to narrowing of artery. On the contrary, MI is an emergency and much more serious. It suddenly happens in a few minutes when the oxygen supply is blocked, and results in myocardial cell death in a few hours. Therefore, the prevention and reconstruction of the occluded artery is the key factor for MI.^[3]^

The treatment of statins for the prevention of recurrent MI has been demonstrated in several randomized controlled trials.^[4]^ Statins is an inhibitor of hydroxymethylglutaryl-CoA reductase, and it is identified to have pleiotropic effects, such as anti-inflammatory and antithrombotic properties and antioxidant effects.^[5–7]^ Therefore, statins are regarded as an important agent for the prevention of MI. Some studies showed that statin pretreatment is associated with a significant reduction in MI. However, other clinical studies showed that early use of statin did not reduce the occurrence of MI.^[8,9]^ Therefore, a more comprehensive analysis of benefits of statin for MI is needed. Thus, we performed a meta-analysis of 18 randomized controlled trials to reevaluate the efficacy of statin treatment to prevent MI in patients.

## 2 Material and methods

### 2.1 Publication search

We obtained relevant randomized controlled trials from Pubmed, Embase, and Chinese biomedicine database that were treated with statin and MI. For the computer searches, we used the following key words: “statin,” “Atorvastatin,” “Rosuvastatin,” “Pravastatin,” “Myocardial infarction,” or “MI,” in the title or abstract, and was limited by “clinical trials, randomized controlled trial-” published in English between 2005 and 2017. Studies contained available data that showed the association of statin treatment in MI. Among the studies with overlapping data published by the same author, only the complete study was included in this meta-analysis. Furthermore, included studies had to show their results as an odds ratio (OR) and 95% confidence interval (95% CI).

### 2.2 Data extraction and classification

For each study characteristics, data were extracted, including the first author, publication year, type of statin, type of study design, sample characteristics, sample size and OR, and risk estimates with corresponding 95% CI.

### 2.3 Statistical analysis

The measure of effect of interest is the OR and the corresponding 95% CI. We showed all results as OR for simplicity and quantified the association of statin treatment in MI, using random-effects models of OR comparing the highest with the lowest category. The summary OR estimates were obtained from random effects models.^[10]^

For all analyses, *P* < .05 were considered significant. Publication bias was assessed by a Begg-adjusted rank correlation test (funnel plot method) and Egger linear regression asymmetry test.^[11]^ All mate-analyses were carried out using Stata software (version 9.0; Stata Corporation, College Station, TX).

### 2.4 Ethical approval

Ethical approval was waived or not necessary. Because we did not make any clinical research in this manuscript, we just collected the data from available publications.

## 3 Results

### 3.1 Characteristics of studies for meta-analysis

A total of 18 publications were identified for inclusion statin in the MI (Table 1).^[12–27]^ Among the 18 studies, 10 described treatment with atorvastatin, 5 with rosuvastatin, and the other 3 treatment with pravastatin. All studies compared a statin with placebo. Of the18 placebo-controlled studies, 16 showed that statins were effective in reducing the incidence of MI.

**Table 1:**
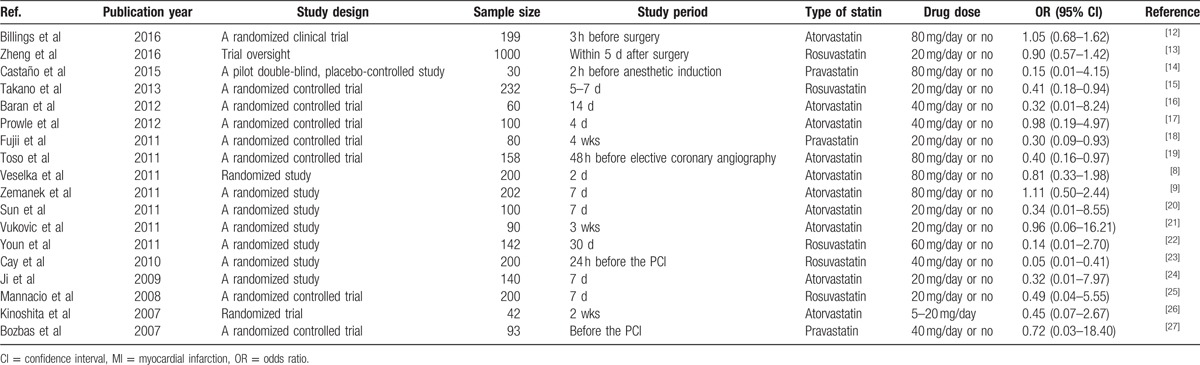
The distribution and ORs (95% CI) for studies on MI and statin.

### 3.2 Statin and MI

**Figure 1:**
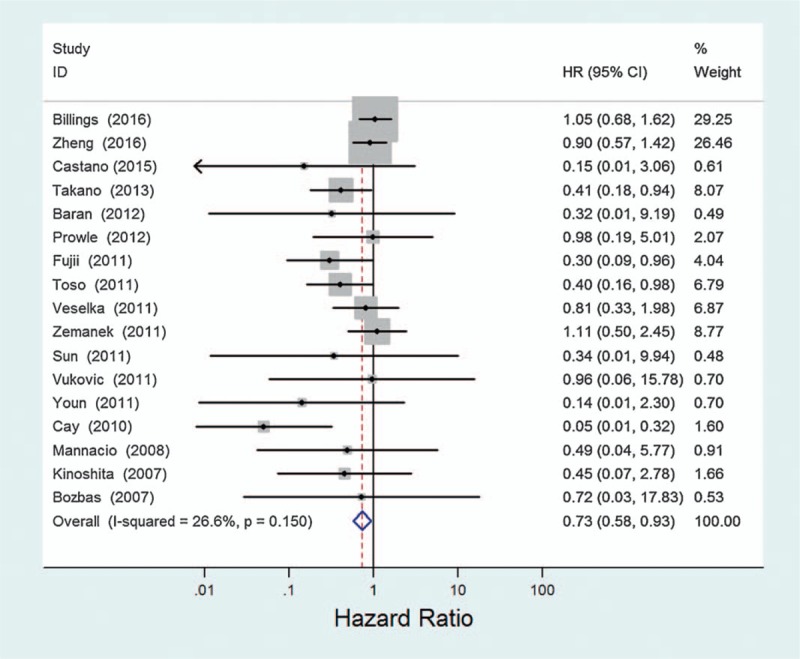
Estimated odds ratio (OR) of risk for MI under statin therapy.

The association of statin treatment of MI was identified in 18 studies, including comparisons of atorvastatin versus placebo, rosuvastatin versus Placebo, and pravastatin versus placebo (Table 1). Pooled estimates showed a statistically significant 27% reduction in the risk of MI with statin (OR = 0.73, 95% CI 0.58–0.93, *P* = .010) (Fig. 1, Table 2). These data indicate that statin was associated with a reduction in MI.

**Table 2:**
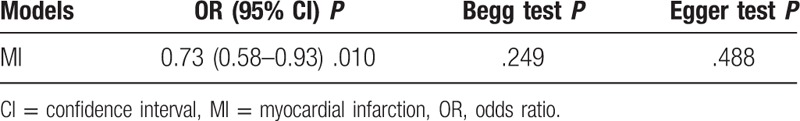
Summary ORs and 95% CI for statin and MI.

## 4 Discussion

Our meta-analysis suggests that statins have demonstrated efficacy in treating or reducing the risk of MI. Statins have a little protective effect for MI, with a 27% lower risk in MI. The intense inhibition of hydroxymethylglutaryl-CoA reductase function precipitated by statin therapy can lead to inhibition of buildup of plaque.

Although the exact mechanisms underlying the early protective effects of statin in cardiovascular events remain undetermined, the statin still contains pleiotropic effect, which includes anti-inflammation, anti-platelet aggregation, and plaque stability.^[28,29]^ Studies suggested that a reduction of MI injury after statin treatment is associated with attenuated inflammatory response.^[30]^ This may be the reason that patient with acute coronary syndromes may benefit most from statins therapy before MI. In addition, animal studies also showed that cardioprotection of statin reloading before ischemia can be restored.^[31]^ This suggested that statin treatment is needed to reach the desired pleiotropic effects.

In summary, our meta-analysis provided some support for the hypothesis that statins have demonstrated efficacy in treating or reducing the risk of MI. However, the number of studies is not enough and we just analyze the data of OR. Future well-designed, large studies might be necessary and should consider the interrelations between different statins.

## 5 Author contributions

**Data curation:** H. Zhang, Y. Zhang, Y. Wang.

**Software:** L. Yin, L. Zhang.

**Writing – original draft:** X. Han.

**Writing – review & editing:** B. Li.
